# Quercetin Reduces Antinociceptive but Not the Anti-Inflammatory Effects of Indomethacin, Ketorolac, and Celecoxib in Rats with Gout-like Pain

**DOI:** 10.3390/molecules30153196

**Published:** 2025-07-30

**Authors:** José Aviles-Herrera, Guadalupe Esther Ángeles-López, Myrna Déciga-Campos, María Eva González-Trujano, Gabriel Fernando Moreno-Pérez, Ricardo Reyes-Chilpa, Irma Romero, Amalia Alejo-Martínez, Rosa Ventura-Martínez

**Affiliations:** 1 Departamento de Farmacología, Facultad de Medicina, Universidad Nacional Autónoma de México (UNAM), Ciudad de México 04510, Mexico; joseaviles@comunidad.unam.mx; 2 Sección de Estudios de Posgrado e Investigación, Escuela Superior de Medicina, Instituto Politécnico Nacional (IPN), Ciudad de México 11340, Mexico; mdeciga@ipn.mx; 3 Laboratorio de Neurofarmacología de Productos Naturales, Dirección de Investigaciones en Neurociencias, Instituto Nacional de Psiquiatría “Ramón de la Fuente Muñiz”, Ciudad de México 14370, Mexico; evag@inprf.gob.mx (M.E.G.-T.); mmspoodles@gmail.com (G.F.M.-P.); 4 Instituto de Química, Universidad Nacional Autónoma de México (UNAM), Ciudad de México 04510, Mexico; chilpa@unam.mx; 5 Departamento de Bioquímica, Facultad de Medicina, Universidad Nacional Autónoma de México (UNAM), Ciudad de México 04510, Mexico; irma@bq.unam.mx; 6 Laboratorio 7 de Dolor y Analgesia, Departamento de Farmacobiología, Centro de Investigación y de Estudios Avanzados (CINVESTAV), Sede Sur, Ciudad de México 14330, Mexico; amy.almart@gmail.com

**Keywords:** gout pain, infra-additive interaction, natural products, NSAIDs, quercetin

## Abstract

The objective of this study was to determine the pharmacological interaction of some common NSAIDs in the presence of quercetin (QUER). Indomethacin (IND), ketorolac (KET), or celecoxib (CEL) were assessed alone and in combination with QUER using experimental gout-arthritic pain and the carrageenan-induced edema test in rats to evaluate their antinociceptive and anti-inflammatory effects, respectively. The antinociceptive effect of each NSAID was also analyzed after the repeated administration of QUER for 10 days. Molecular docking analysis on COX-1/COX-2 with each drug was explored to analyze the pharmacological interaction. QUER produced minimal antinociceptive or anti-inflammatory effects on experimental gout-arthritic pain or on the carrageenan-induced edema in rats. Additionally, QUER reduced the antinociceptive effect of NSAIDs, mainly those COX-1 inhibitors (IND and KET), when they were combined. However, QUER did not modify the anti-inflammatory effect of these COX-1 inhibitors and slightly improved the anti-inflammatory effect of the COX-2 inhibitor (CEL). According to the docking analysis, COX-1 and COX-2 are likely implicated in these pharmacological interactions. In conclusion, QUER, a known bioactive natural product, may alter the antinociceptive efficacy of NSAIDs commonly used to relieve gout-like pain and suggests not using them together to prevent a negative therapeutic interaction in this effect.

## 1. Introduction

**Figure 1:**
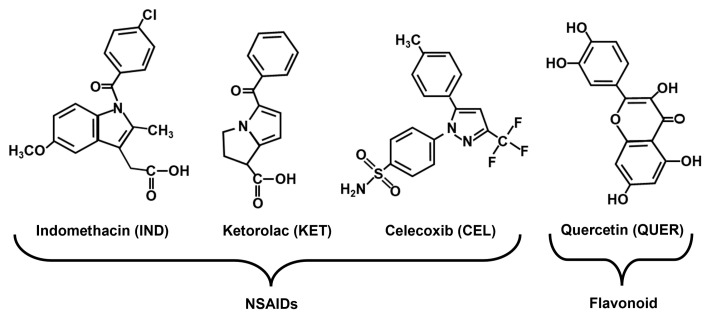
Chemical structures of some non-steroidal anti-inflammatory drugs (NSAIDs), suchas indomethacin (IND), ketorolac (KET), and celecoxib (CEL), as well as quercetin (QUER), a flavonoid.

Pain is an unpleasant sensation and one of the major reasons for medical appointments worldwide [1]. Pain can be caused by several reasons, but diseases affecting the musculoskeletal system, such as arthritis, are among the leading causes of chronic pain [2]. Non-steroidal anti-inflammatory drugs (NSAIDs) are used to treat pain, especially inflammatory pain as it is presented in patients with arthritic diseases. The analgesic and anti-inflammatory effects of NSAIDs are due to the inhibition of cyclooxygenase (COX), which is responsible for the synthesis of prostaglandins. There are two isoforms, COX-1 and COX-2, and it is well known that the inhibition of COX-2 is responsible for the pharmacological effects of NSAIDs, while the inhibition of COX-1 is responsible for their adverse effects, such as gastrointestinal, renal, and cardiovascular issues. NSAIDs are classified according to their capacity of inhibition by each isoform, determined in in vitro assays. In this sense, some NSAIDs inhibit COX-1 or COX-2 more selectively, such as ketorolac (KET) and celecoxib (CEL), respectively (Figure 1). Others inhibit both COX isoforms in a non-selective manner, such as indomethacin (IND) (Figure 1). However, in general, the use of NSAIDs for prolonged periods is not recommended due to their adverse effects [3].

Relevant alternatives for pain treatment are natural products, which are continuously used among people to improve health conditions [4]. Quercetin (QUER) is a well-known flavonoid that possesses antioxidant, antinociceptive [5], and anti-inflammatory [6,7] properties (Figure 1). This flavonoid is one of the most abundant compounds in foods such as apples, blackberries, grapes, onions, broccoli, and kale, as well as in beverages such as green tea, black tea, and red wine [8]. Currently, QUER is sold as a dietary supplement with the idea that, being of natural origin, it does not produce adverse effects or negative drug interactions [9].

Drug synergism can be expected when two or more drugs that act via different mechanisms of action are combined. The pharmacological interaction of natural products in combination with NSAIDs has been described in the case of hesperidin, methyl eugenol, or rutin with ketorolac or diclofenac. Hesperidin causes various types of interactions in a dose-dependent manner, including additive and potentiating synergism, but also antagonism in the PIFIR model, mediated by the participation of TRPV1 receptors [10]. On the other hand, methyl eugenol increased the antinociceptive effect of ketorolac in the formalin test in mice, mediated by the blockade of Nav1.7 channel overexpression and the activation of GABAA receptors [11]. Rutin also produced synergism on the antinociceptive effect of ketorolac and diclofenac in the acetic-acid-induced writhing test, mediated by the effect of rutin on the opioidergic system and the downregulation of p38-MAPK and NF-κB [12].

In general, a good pharmacological strategy for the use of a combination of drugs would be to increase the therapeutic effects of drugs and/or to reduce their adverse effects. However, they must be administered carefully since unwanted or toxic effects could also be potentiated. On the other hand, a combination of drugs can also produce infra-additive effects when they act via similar mechanisms of action. QUER is a flavonoid that has demonstrated antinociceptive and/or anti-inflammatory properties in different pain models [13,14,15]; however, there is scarce information exploring the pharmacological interaction of specific doses of QUER with those of commonly used NSAIDs to relieve pain. As far as we know, there is only a preliminary report by our group where QUER significantly decreased the antinociceptive response of the NSAID diclofenac [16]. Therefore, this study aimed to determine the pharmacological interaction of the bioactive flavonoid QUER based on the antinociceptive and anti-inflammatory effects of COX-1 and COX-2 NSAIDs such as KET, IND, and CEL, respectively, in a gout arthritic pain model and carrageenan-induced edema in rats using the pharmacometric strategy of the surface of synergistic interaction (SSI) and molecular docking analysis of COX-1 and COX-2.

## 2. Results

### 2.1. In Vivo Study

#### 2.1.1. Antinociceptive Effect After Individual Administration of QUER, IND, KET, or CEL in the PIFIR Model

**Figure 2:**
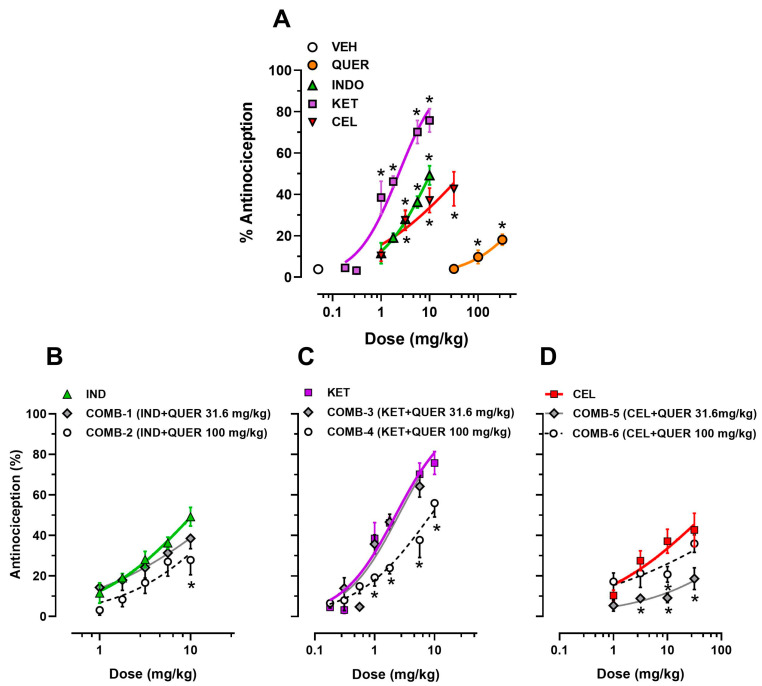
(**A**) Dose-response curves (DRCs) of the antinociceptive effect of quercetin (QUER, 31.6–316 mg/kg, ip), indomethacin (IND, 1–10 mg/kg, po), ketorolac (KET, 0.31–10 mg/kg, po), and celecoxib (CEL, 1.0–31.6 mg/kg, po) in individual administration. Each point represents the mean ± S.E.M. of six animals. * *p* < 0.05 vs. VEH group. (**B**) Antinociceptive effect induced by IND (1–10 mg/kg) in the absence and the presence of 31.6 mg/kg QUER (COMB-1) or 100 mg/kg QUER (COMB-2). (**C**) Antinociceptive effect induced by KET (0.31–10 mg/kg) in the absence and the presence of 31.6 mg/kg QUER (COMB-3) or 100 mg/kg QUER (COMB-4). (**D**) Antinociceptive effect induced by CEL (0.31–31.6 mg/kg) in the absence and the presence of 31.6 mg/kg QUER (COMB-5) or 100 mg/kg QUER (COMB-6). Each point represents the mean ± S.E.M. of the six animals per group. In this analysis, the effect of the individual administration of QUER was not considered. * *p* < 0.05 vs. effect induced by the corresponding NSAIDs in individual administration, two-way ANOVA followed by Tukey’s test.

The intra-articular administration of uric acid induced a progressive diminution of the functionality of animals. When rats developed total dysfunction of the injured paw, established as the % FI = zero value (approximately 2 h after uric acid administration), treatments were administered. The recovery of the dysfunction was considered the antinociceptive effect. Under these conditions, QUER at 31.6 mg/kg did not induce an antinociceptive effect, while 100 mg/kg (12.8 ± 2.0%) or 316 mg/kg (18.1 ± 2.8%) produced a minimal antinociceptive response reaching a significant difference compared to the vehicle (VEH) group (3.2 ± 1.3%) (*p* = 0.0002; F(3, 20) = 10.58). Similarly, the NSAIDs promoted a significant antinociceptive response vs. the VEH group, such as IND at 3.16 mg/kg (28.1 ± 4.0%), 5.6 mg/kg (36.4 ± 2.9%), or 10 mg/kg (49.2 ± 4.6%), KET at 1 mg/kg (38.5 ± 7.9%), 1.78 (46.3 ± 2.7%), 5.6 (70.2 ± 5.6%), or 10 mg/kg (75.8 ± 5.6%), and CEL at 3.16 mg/kg (27.5 ± 4.8%), 10 mg/kg (37.1 ± 5.9%), or 31.6 mg/kg (42.7 ± 8.3%) (*p* < 0.0001; F(16, 91) = 21.03) (Figure 2A).

#### 2.1.2. Antinociceptive Effect of NSAIDs in the Presence of the Acute Administration of QUER

QUER at 31.6 mg/kg did not modify the antinociceptive effect of any explored dose of IND, while QUER at 100 mg/kg significantly diminished the antinociceptive effect of the highest dose of IND (10 mg/kg) (49.2 ± 4.6% vs. 27.9 ± 7%, *p* < 0.0001; F(4, 76) = 15.78) (Figure 2B).

QUER at 31.6 mg/kg did not modify the antinociceptive effect of KET (Figure 2C), while QUER at 100 mg/kg significantly diminished the antinociceptive effect of KET at doses of 1.0 mg/kg (38.5 ± 7.9 to 19.26 ± 4.25%), 1.78 mg/kg (46.25 ± 2.7 to 23.94 ± 2.87%), 5.6 mg/kg (70.18 ± 5.6 to 37.74 ± 8.6%), and 10 mg/kg (75.8 ± 5.65 to 56.0 ± 6.9%) (*p* < 0.0001; F(6, 107) = 78.525) (Figure 2C).

Finally, QUER at 31.6 mg/kg significantly reduced the antinociceptive effect of CEL evaluated at 3.16 mg/kg (27.53 ± 4.8 vs. 8.92 ± 1.9%), 10 mg/kg (37.13 ± 5.9 vs. 9.15 ± 2.2%), and 31.6 mg/kg (42.69 ± 8.29 vs. 14.85 ± 4.5%) (Figure 2D). However, when QUER was increased to 100 mg/kg, only the antinociceptive effect of CEL at 10 mg/kg was diminished (37.13 ± 5.9% vs. 20.77 ± 3.8%) (*p* < 0.0001; F(3, 66) = 9.38) (Figure 2D).

#### 2.1.3. Analysis of Interaction of IND, KET, or CEL in Combination with QUER

To identify the type of interaction produced by NSAIDs in the presence of QUER, the SSI analysis was performed by building a group of three-dimensional graphs for each combination [17].

**Figure 3:**
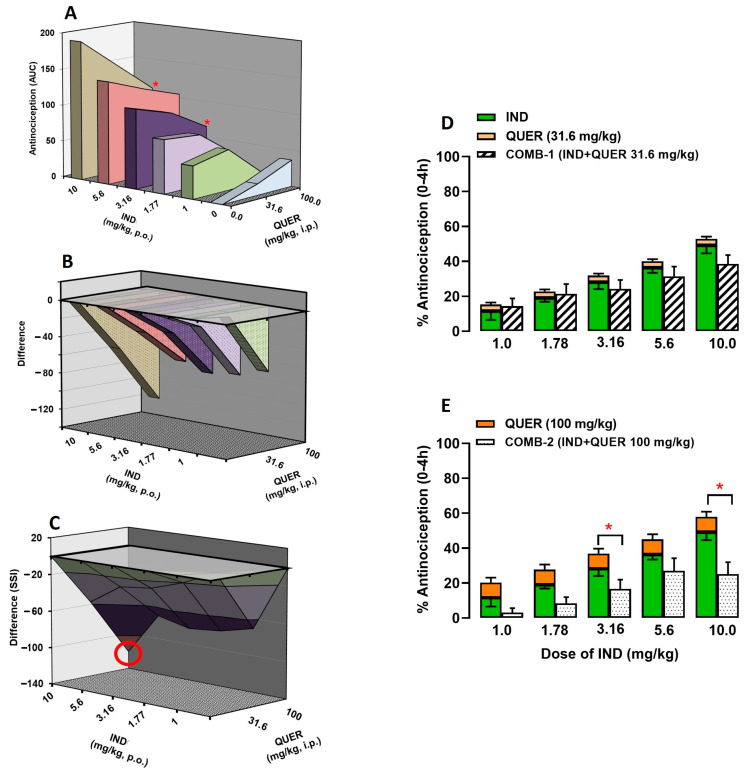
(**A**–**C**) Three-dimensional graphics from the analysis of the interaction of the antinociceptive effect of the combination of indomethacin (IND) with QUER. (**A**) Doses of IND (Y-axis) and QUER (X-axis) and their antinociceptive effects in individual administration or in combination (Z-axis). (**B**) Subtraction of the antinociceptive effect of each combination minus the individual effect of IND and QUER. (**C**) Surface of synergistic interaction of the antinociceptive effect of indomethacin (IND) with QUER. The red circle indicates the combination that induced the maximum antinociceptive interaction (MAI) of this combination. (**D**,**E**) Two-dimensional graphics showing the individual effects of IND and QUER at 31.6 mg/kg (**D**) or 100 mg/kg (**E**). In both, the first bar shows the antinociceptive effect of IND and QUER in individual administration and represents the sum of the individual effects of the drugs (expected effect), while the second bar (with a white background) represents the antinociceptive effect obtained experimentally with the respective combination. * *p* < 0.05 vs. expected effect (addition of the antinociceptive effect of IND and QUER in individual administration). Two-way ANOVA followed by Tukey’s test.

Figure 3 shows the interaction of IND + QUER (*p* = 0.99; F(12, 102) = 0.26). In Figure 3A, IND doses are plotted on the “X” axis; the antinociceptive effect of the treatments is shown on the “Y” axis, while QUER doses are presented on the “Z” axis. Each intermediate point represents the antinociceptive effect obtained for the corresponding combination. In the first 3-D graphic, only the combinations of QUER at 100 mg/kg with IND at 3.16 mg/kg (178.9 ± 23.4 vs. 104.8 ± 27.8 au) and 10 mg/kg (228.8 ± 30.1 vs. 108.1 ± 28.4 au) showed a significant difference between the sum of the individual effects of each drug and the obtained effects (*p* < 0.0001; F(4, 102) = 17.26). Figure 3B represents the result of subtracting the effects produced by each drug when administered individually from the effect obtained with the respective combination. The “Y” axis shows the “difference” between these algebraic considerations. In the second 3-D graphic, all points were found below line 0, indicating an infra-additive interaction. Finally, Figure 3C shows the SSI of the IND + QUER combination obtained by joining the points of the second 3-D graph. In this last 3-D graphic, the combination that induced the maximum antinociceptive interaction (MAI) was IND (10) + QUER (100), which induced an effect 52% lower in comparison to the sum of their individual effects (see the data highlighted in a red circle in Figure 3C).

The bar graphs show the individual antinociceptive effects of IND and QUER at 31.6 mg/kg (Figure 3D) or 100 mg/kg (Figure 3E). The first bar represents the expected effect of the IND + QUER combination, which is the sum of the individual effects of the drugs at the corresponding doses. The second bar (with a white background) represents the experimentally obtained antinociceptive effect (obtained effect) with the combination of IND + QUER at the corresponding doses. Only the combinations of QUER 100 mg/kg with IND 3.16 and 10 mg/kg showed a significant difference between the expected and obtained effects.

**Figure 4:**
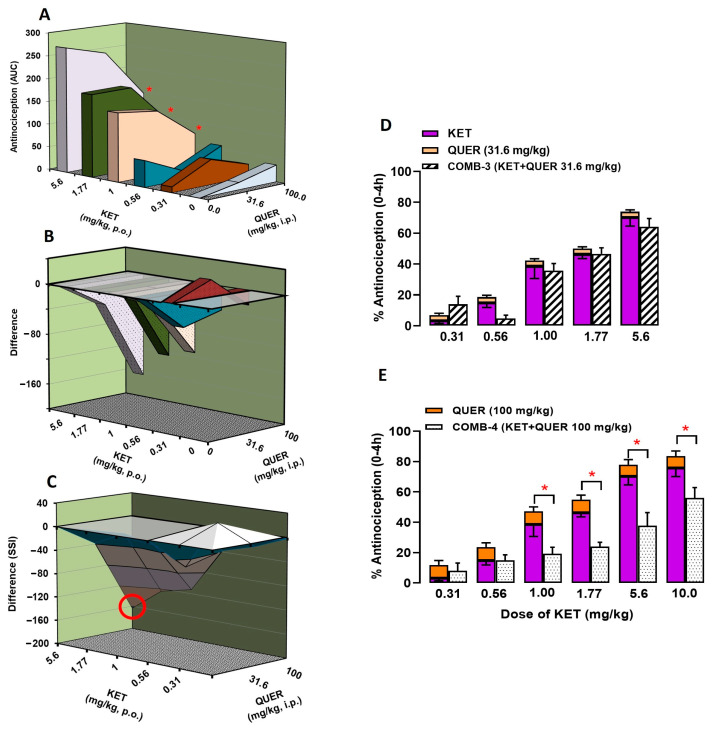
(**A**–**C**) Three-dimensional graphics from the analysis of the interaction of the antinociceptive effect of the combination of ketorolac (KET) with QUER. (**A**) Doses of KET (Y-axis) and QUER (X-axis) and their antinociceptive effects in individual administration or in combination (Z-axis). (**B**) Subtraction of the antinociceptive effect of each combination minus the individual effect of KET and QUER. (**C**) Surface of synergistic interaction of the antinociceptive effect of ketorolac (KET) with QUER. The red circle indicates the combination that induced the maximum antinociceptive interaction (MAI) of this combination. (**D**,**E**) Two-dimensional graphics showing the individual effects of KET and QUER at 31.6 mg/kg (**D**) or 100 mg/kg (**E**). In both, the first bar shows the antinociceptive effect of KET and QUER in individual administration and represents the sum of the individual effects of the drugs (expected effect), while the second bar (with a white background) represents the antinociceptive effect obtained experimentally with the respective combination. * *p* < 0.05 vs. expected effect (addition of the antinociceptive effect of KET and QUER in individual administration). Two-way ANOVA followed by Tukey’s test.

Figure 4 shows the interaction of KET + QUER (*p* = 0.19; F(15, 125) = 1.33). In Figure 4A, KET doses are plotted on the “X” axis, with QUER doses on the “Z” axis, and the antinociceptive effect of the treatments is shown on the “Y” axis. Each point in between represents the antinociceptive effect obtained for the corresponding combination. In this 3-D graph, the combinations of QUER at 100 mg/kg with four doses of KET, such as 1 mg/kg (187.3 ± 46.3 vs. 74.63 ± 16.51 au), 1.77 mg/kg (217.3 ± 23.7 vs. 92.8 ± 11.1 au), 5.6 mg/kg (310.0 ± 33.8 vs. 146.3 ± 33.3 au), and 10 mg/kg (331.8 ± 34.2 vs. 217.0 ± 26.7 au), showed a significant difference between the sum of the individual effects of each drug and the obtained effect (*p* < 0.0001; F(5, 125) = 59.07). Similarly to the IND + QUER combination, Figure 4B represents the result of subtracting the effects produced by KET and QUER in individual administration minus the effect obtained with the respective combination, where the “Y” axis shows the “difference” of these algebraic considerations. In this graph almost all points were found below line 0, indicating an infra-additive interaction, except in the combination of KET (0.31) + QUER (31.6), where an additive interaction was observed (53.9 ± 20.2 vs. 27.9 ± 10.9 au). In the SSI (Figure 4C) of the KET + QUER combination obtained by joining the points of the previous graph, the combination that induce the MAI was KET (5.6) + QUER (100), which induced an effect 52.5% lower than the sum of their individual effects (see data highlighted in a circle in Figure 4C).

The infra-additive interaction of the KET + QUER combination was confirmed by two bar graphs, which showed the individual effects of KET and QUER at 31.6 mg/kg (Figure 4D) or 100 mg/kg (Figure 4E). In both, the first bar shows the antinociceptive effect of KET and QUER when administered individually at the corresponding doses. Thus, the first bar represents the expected effect of the KET + QUER combination, which is the sum of the individual effects of the drugs, while the second bar (with a white background) represents the experimentally obtained antinociceptive effect of the KET + QUER combination. Indeed, four combinations of QUER at 100 mg/kg with KET (1.0, 1.77, 5.6, or 10 mg/kg) showed significant differences between the expected and obtained effects.

**Figure 5:**
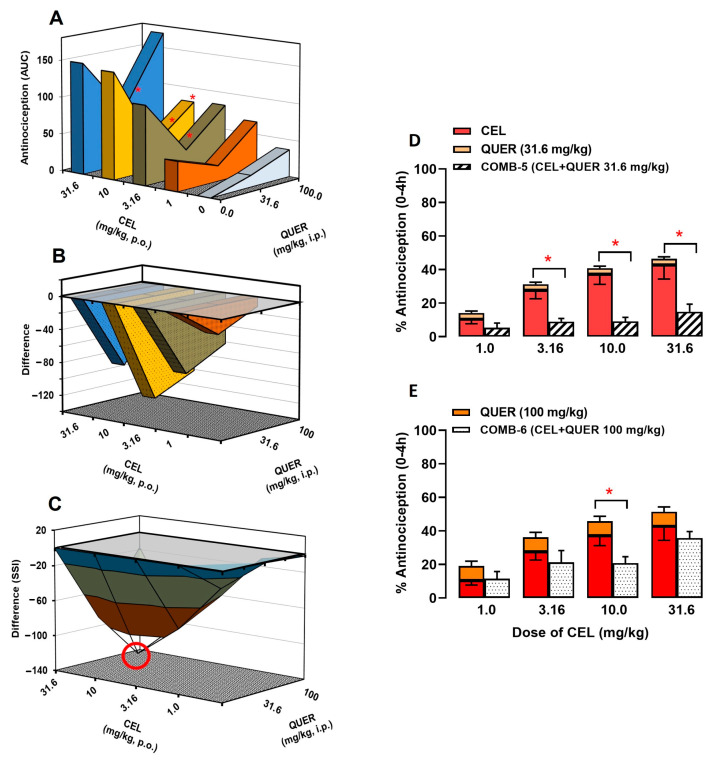
(**A**–**C**) Three-dimensional graphics from the analysis of the interaction of the antinociceptive effect of the combination of celecoxib (CEL) with QUER. (**A**) Doses of CEL (Y-axis) and QUER (X-axis) and their antinociceptive effects in individual administration or in combination (Z-axis). (**B**) Subtraction of the antinociceptive effect of each combination minus the individual effect of CEL and QUER. (**C**) Surface of synergistic interaction of the antinociceptive effect of celecoxib (CEL) with QUER. The red circle indicates the combination that induced the maximum antinociceptive interaction (MAI) of this combination. (**D**,**E**) Two-dimensional graphics showing the individual effects of CEL and QUER at 31.6 mg/kg (**D**) or 100 mg/kg (**E**). In both, the first bar shows the antinociceptive effect of CEL and QUER in individual administration and represents the sum of the individual effects of the drugs (expected effect), while the second bar (with a white background) represents the antinociceptive effect obtained experimentally with the respective combination. * *p* < 0.05 vs. expected effect (addition of the antinociceptive effect of CEL and QUER in individual administration). Two-way ANOVA followed by Tukey’s test.

Figure 5 shows the interaction of CEL + QUER (*p* = 0.76; F(12, 111) = 0.68), where in Figure 5A, the doses of CEL are represented in the “X” axis, with the doses of QUER on the “Z” axis, and the antinociceptive effect of treatments is shown in the “Y” axis. Each intermediate point represents the antinociceptive effect obtained for the corresponding combination. In this 3-D graphic, three combinations of QUER at 31.6 mg/kg with CEL at 3.16 mg/kg (36.6 ± 7.5 vs. 122.25 ± 23.8 au), 10 mg/kg (35.5 ± 9.1 vs. 159.5 ± 27.9 au), and 31.6 mg/kg (75.3 ± 20.8 vs. 165.2 ± 31.6, *p* < 0.0001) achieved lower effects than the sum of the effects of the drugs individually administered, while only the low dose of CEL (10 mg/kg) combined with QUER at 100 mg/kg achieved lower effects in comparison to the sum of the effects of the drugs individually administered (80.48 ± 15.0 to 181.9 ± 25.3 au) (*p* < 0.0001; F(3, 111) = 15.97). Figure 5B represents the result from the subtraction of the effects produced by CEL and QUER in individual administration minus the obtained effect with the respective combination, where the “Y” axis shows the “difference” of these algebraic considerations. In this graphic, all points were found below line 0, indicating an infra-additive interaction. In the analyses of SSI, the combination with the major infra-additive interaction was CEL (10) + QUER (31.6), which induced an effect 78% lower than the sum of their individual effects (see data highlighted in a circle in Figure 5C).

The infra-additive interaction from the combination of CEL + QUER was confirmed with two bar charts, which showed the individual effects of CEL and QUER at 31.6 mg/kg (Figure 5D) or at 100 mg/kg (Figure 5E). In both, the first bar shows the antinociceptive effect of CEL and QUER in individual administration and represents the expected effect of the combination of CEL with QUER that is the sum of the individual effects of the drugs, while the second bar (with a white background) represents the antinociceptive effect obtained experimentally for the combination of CEL + QUER. In this case, three combinations of QUER at 31.6 mg/kg with CEL (3.16, 10, or 31.6 mg/kg) and one combination of QUER at 100 mg/kg with CEL 10 mg/kg showed significant differences between the expected effects and the obtained effects.

It is important to mention that, according to these results, the greatest infra-additive antinociceptive interactions were obtained with the combinations IND (10) + QUER (100), KET (5.6) + QUER (100), and CEL (10) + QUER (100) (Figure 3, Figure 4 and Figure 5, respectively). Thus, the subsequent experiments to evaluate the anti-inflammatory effects of individual and combined administration were explored using such infra-additive interactions (MAI).

#### 2.1.4. Effect of the Repeated Administration of QUER on the Antinociceptive Effect of IND, KET, or CEL in the PIFIR Model

**Figure 6:**
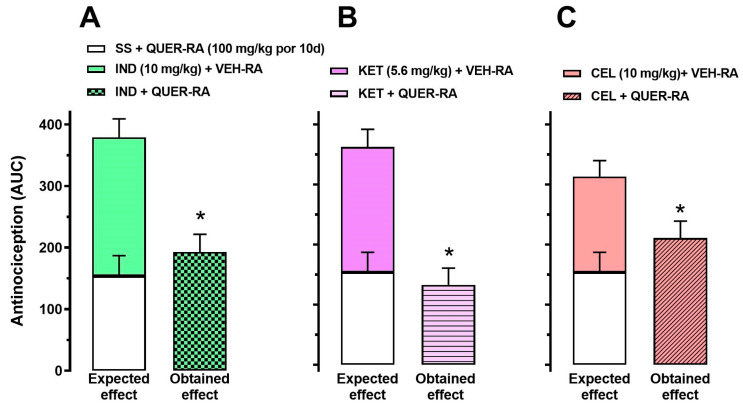
Area under the curve from the temporal courses of the effect of repeated administration (RA) of QUER on the antinociception induced by IND (**A**), KET (**B**), or CEL (**C**). In this protocol, the animals were pretreated with VEH (VEH-RA) or QUER 100 mg/kg/day for 10 days (QUER-RA). The administration of NSAIDs or saline solution (SS) was performed on the 11th day, 2 h after the intraarticular administration of uric acid. Each bar represents the mean ± SEM of six animals. The expected effect is the sum of SS + QUER-RA with the respective NSAID + QUER-RA, while the obtained effect is the experimental effect of each combination. * *p* < 0.05, unpaired two-tailed Student’s *t*-test.

To determine if the repeated administration of QUER modified the antinociceptive effect of the explored NSAIDs, a dosage of QUER (100 mg/kg) or VEH was administered daily for 10 days (RA). On the 11th day, saline solution (SS, 0.9% NaCl), IND (10 mg/kg), KET (5.6 mg/kg), or CEL (10 mg/kg) was administered to the rats with a previous intraarticular injection of uric acid (2 h before) and evaluated in a temporal course, recorded every 30 min for 4 h (Table 1). Interestingly, under these experimental conditions, animals with repeated SS + QUER-RA did not reach the total disfunction with uric acid in comparison to animals with QUER (100 mg/kg) in acute administration, which reached total disfunction 2.5 h after uric acid injection (Figure S1 of Supplementary Materials). Nevertheless, the effect produced by each NSAID (IND, KET, or CEL) in rats treated with vehicle repeated for 10 days, VEH-RA, did not show a difference in comparison to NSAIDs with the previous repeated administration of QUER-RA (100 mg/kg) (Table 1 and Figure 6).

The analysis of the AUC of each individual treatment in comparison to the antinociceptive effect of QUER in repeated administration (SS + QUER-RA) showed a significantly lower effect in the treatment of IND + QUER-RA (*p* = 0.0014; t = 4.358, df = 10), KET + QUER-RA (*p* = 0.0003; t = 5.462, df = 10), and CEL + QUER-RA (*p* = 0.032; t = 2.491, df = 10) (Figure 6A, Figure 6B and Figure 6C respectively) (observed effect) in comparison with the theorical sum of the individual effects of each component (expected effect) (Figure 6).

#### 2.1.5. Anti-Inflammatory Effect of NSAIDs in Combination with QUER in the Carrageenan Test

Carrageenan-induced edema in the paws of rats receiving VEH was evaluated in a temporal course registered at 1, 2, 3, 4, 5, and 6 h to determine a significant inflammatory effect (*p* < 0.0001; F(6, 280) = 107.2) (Table 2). The individual administration of the highest dose of QUER (100 mg/kg) did not modify the paw edema induced by carrageenan, whereas the individual administration of IND at 10 mg/kg produced a significant diminution of the paw edema (anti-inflammatory effect) starting at 2 h and remaining until 6 h of the register compared to the VEH group (*p* < 0.0001; F(6, 105) = 34.1). This effect of IND alone did not change at any time in the presence of QUER (*p* = 0.86; F(6, 70) = 0.4135) (Table 2). In the same way, the individual administration of KET also induced a significant diminution of the paw edema (anti-inflammatory effect) from 2 h until 6 h compared to the VEH group (*p* < 0.0001; F(6, 105) = 48.26), which was not modified in the presence of QUER (*p* = 0.96; F(6, 70) = 0.2223) (Table 2).

Finally, CEL at 10 mg/kg significantly diminished the paw edema (anti-inflammatory effect) starting at 2 and 3 h after its administration compared to the vehicle (VEH) group. In contrast, the combination of CEL + QUER induced an anti-inflammatory effect from 2 h that remained until 6 h of recording compared to the vehicle (VEH) group (*p* < 0.0001; F(6, 105) = 44.07) (Table 2), although, the effect of CEL alone was not significant different from CEL + QUER at any time (*p* = 0.67; F(6, 70) = 0.677). The data represent carrageenan-induced edema, so the greater the anti-inflammatory effect, the lower the percentage of paw edema observed (Table 2).

### 2.2. In Silico Study

#### 2.2.1. COX-1–Ligand Interactions

**Figure 7:**
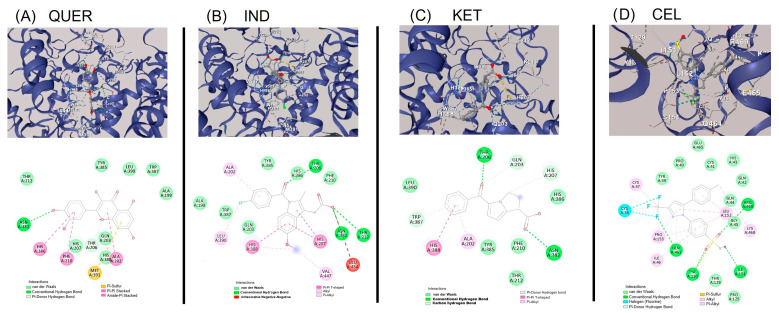
Molecular docking analysis with COX-1 enzyme, 3D binding poses in the COX-1 active site showing hydrogen bonds and π-π interactions, and 2D interaction map highlighting interactions of (**A**) quercetin (QUER), (**B**) indomethacin (IND), (**C**) ketorolac (KET), and (**D**) celecoxib (CEL).

QUER demonstrated the strongest binding affinity towards COX-1, with a docking score of −8.4 kcal/mol. It formed multiple hydrogen bonds with THR206, HIS204, LYS211, and GLU454, supported by π-π stacking with TYR385 and TRP387. Additional van der Waals interactions were observed with ALA199, ALA202, VAL291, ASN382, HIS386, and LEU390, contributing to ligand stabilization within the active site (Figure 7A). IND also displayed high affinity (−8.4 kcal/mol), forming hydrogen bonds with GLU290, LYS211, THR212, and GLN289, as well as π-π interactions with TYR385 and TRP387. Hydrophobic and van der Waals contacts involved ALA202, HIS207, VAL291, LEU294, LEU298, and PHE409, establishing a robust binding configuration (Figure 7B). KET, with a docking score of −8.1 kcal/mol, showed a dense interaction profile. It established hydrogen bonds with GLN203, LYS211, and GLU454, along with π-π stacking with TYR385 and TRP387. Van der Waals interactions with ALA199, PHE200, VAL291, ASN382, and LEU390 further supported its placement within the binding pocket (Figure 7C). CEL exhibited the lowest binding affinity for COX-1 (−7.7 kcal/mol) yet maintained stable interactions via hydrogen bonds with GLU524 and ARG120 and π-π interactions with TRP77 and TYR64. Its interaction pattern also included van der Waals contacts with residues such as CYS41, ARG79, PRO84, and PHE470, suggesting moderate stabilization (Figure 7D).

#### 2.2.2. COX-2 Ligand Interactions

**Figure 8:**
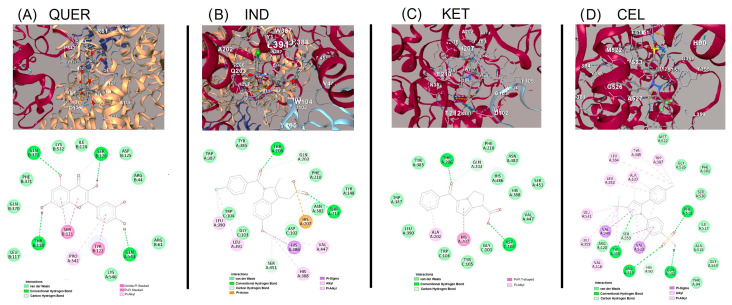
Molecular docking analysis with COX-2 enzyme, 3D binding poses in the COX-1 active site showing hydrogen bonds and π-π interactions, and 2D interaction map highlighting interactions of (**A**) quercetin (QUER), (**B**) indomethacin (IND), (**C**) ketorolac (KET), and (**D**) celecoxib (CEL).

QUER, with a docking score of −9.2 kcal/mol, formed hydrogen bonds with THR118, SER119, and ASP125, while van der Waals contacts involved ARG44, TYR122, GLN370, and PHE371. Despite a different binding orientation, π-π stacking with TRP387 was also evident, indicating an alternative but effective interaction mode (Figure 8A). IND (−9.5 kcal/mol) established hydrogen bonds with GLN203, HIS214, and GLN454 and π-π interactions with TYR385, TRP387, and TYR105 (Chain C). Other relevant residues included VAL447, ALA450, and LEU391, contributing to a well-integrated binding profile (Figure 8B). IND and KET exhibited a strong binding score (−9.5 kcal/mol). CEL engaged in hydrogen bonds with SER119, TYR355, and GLU524, with additional π-π stacking observed at TYR385 and TRP387. The ligand was also stabilized by extensive hydrophobic contacts including VAL349, PHE381, LEU472, and PHE529 (Figure 8C). Among the tested compounds, CEL showed the highest binding affinity for COX-2 (−9.6 kcal/mol). It formed multiple hydrogen bonds with GLU290, THR212, GLN454, and ARG222, in addition to π-π stacking with TRP387 and TYR385. Key van der Waals interactions involved residues such as ALA202, ASN382, LEU390, and LEU391, spanning Chains A and C (Figure 8D).

During receptor preparation, all crystallographic water molecules were removed to standardize the docking environment and improve the reliability of pose prediction. Most molecular docking tools, including AutoDock, use an implicit solvent model that does not treat water molecules dynamically. In this context, retaining individual water molecules unless clearly conserved and functionally relevant can interfere with ligand placement and introduce artifacts into binding affinity predictions [18,19]. Although certain structural waters may contribute to ligand recognition via bridging interactions between the protein and the ligand, these are typically identified based on conservation across homologous structures, low B-factors, or repeated hydrogen bonding networks. In this study, no conserved water molecules were detected in the COX-1- or COX-2-binding sites based on structural alignment and a literature review. Therefore, all crystallographic waters were excluded to ensure methodological consistency and avoid non-specific steric or electrostatic interference during docking simulations [20].

The ligand structures were optimized using the MMFF94 (Merck Molecular Force Field), a well-established force field specifically developed by Merck Research Laboratories for modeling small organic molecules of pharmacological relevance. MMFF94 combines empirical parameters with quantum chemical data to achieve high accuracy in reproducing molecular geometries, conformational energetics, torsional profiles, and nonbonding interactions. Its parameterization comprises a wide range of functional groups commonly found in drug-like compounds, making it particularly suitable for ligand preparation in structure-based drug design workflows. Compared to more general-purpose force fields such as UFF or GAFF, MMFF94 has demonstrated superior performance in preserving chemically realistic geometries, which is essential for reliable docking and binding affinity estimation [21].

Furthermore, its implementation is computationally efficient, allowing rapid energy minimization of ligands without compromising conformational accuracy. In this study, a convergence criterion of 0.001 kcal/mol·Å was applied to ensure the stable and low-energy conformations before molecular docking.

## 3. Discussion

It has already been demonstrated that QUER induced an antinociceptive effect in several pain tests, such as the formalin, the hot plate, and the tail-flick tests and even in chronic neuropathic pain models [5,22,23]. In our study, QUER at different doses produced at least a 16% antinociceptive effect in the gouty arthritic pain model in rats that could be without clinical relevance. It is important to mention that the antinociceptive effect of QUER might depend on the type of experimental pain model explored since its effects have been explored in acute nociception when the algogenic stimulus has been induced after the administration of this flavonoid. In this study, QUER was given when the painful process had already established itself using the PIFIR model. This experimental model produces nociceptive pain associated with inflammation induced by uric acid injected into the knee joint of the right hind limb of the rats [24]. The limb dysfunction resembles the difficult-to-treat arthritic pain suffered by patients with gouty arthritis. The PIFIR model has been used to evaluate the antinociceptive effect of NSAID-like drugs and those without anti-inflammatory properties, such as morphine (an opioid drug) [25]. Because it does not directly analyze the anti-inflammatory effect of drugs, we corroborated the anti-inflammatory-like effect of treatments by using the carrageenan edema test to evaluate QUER alone and in combination with COX-1 and COX-2 NSAIDs.

Therapy for inflammatory pain includes the use of several NSAIDs, such as IND and KET, as well as selective inhibitors of COX-2 like CEL, with all of them explored in this investigation. It is well known that the effect produced by NSAIDs is due to the inhibition of COX-1 and/or COX-2 to reduce the synthesis of prostaglandins, which participate in the generation of pain associated with inflammation. The effectiveness of these drugs is variable and depends on the pain stimulus due to the selectivity of each NSAID for the COX isoforms [26]. Nevertheless, other mechanisms may be involved in the antinociceptive effect of NSAIDs; for example, in the case of the antinociceptive effects of IND and KET, both have been involved not only in the inhibition of COX-1 and synthesis of prostaglandins but also in the NO–cGMP pathway at the local level [27,28]. In the antinociceptive effect of KET, the endogenous opioid system has been implicated [29], whereas for CEL, it has been described that diminution in the production of the prostaglandins is mediated by the inhibition of synthesis and downregulating COX-2 expression [30], as well as the inhibition of NF-κB activation in macrophages [31] and the possible involvement of endogenous opioids at the central level [32]. Despite the efficacy of NSAIDs in producing suitable antinociceptive effects with or without the inflammation process, their chronic administration is limited by adverse effects, specifically gastrointestinal lesions and renal or cardiovascular complications [1,3]. It has been reported that flavonoids can act through various molecular mechanisms to produce their anti-inflammatory effects. These include the inhibition of protein kinases, the regulation of transcription factors such as NF-κB, among other signal transduction pathways, and, importantly, the capture of the production of reactive oxygen species (ROS). Consequently, QUER presence plays a direct or indirect impacting role in the modulation of the immune system and inflammatory processes [33]. In particular, and according to both in vivo and in vitro studies, QUER acts as a multitarget ligand that regulates different inflammatory pathways through the activation of antioxidant genes and enzymes. For example, in a dose-dependent manner, it reduces the secretion of TNF-α and IL-1β and inhibits the expression of inducible nitric oxide synthase (iNOS); it also negatively regulates the NF-κB pathway, inhibiting the phosphorylation of IkB-α and preventing the production of proinflammatory cytokines such as TNFα, IL1β, and IL6 [34].

A combination of drugs is based on the premise that two or more drugs that induce the same pharmacological effects through different mechanisms may exhibit a synergistic interaction (additive or supra-additive effect) [35] with a diminution of the adverse effects. Under this premise, it was expected that the combination of QUER with some NSAIDs, all of them considered suitable analgesics and/or anti-inflammatory drugs, might produce a synergistic interaction, mainly with supra-additive effects, since previous combinations of natural products (hesperidin, rutin, or methyleugenol) with some NSAIDs (diclofenac, ketorolac, metamizole, or naproxen, respectively) had reported beneficial responses [10,11,12,36,37]. However, the analysis of SSI carried out in this study presented experimental evidence of nonpositive pharmacological interactions (infra-additive) promoted by the QUER and NSAID (IND, KET, and/or CEL) combination, in which antinociceptive properties involve COX-1 and/or COX-2. These findings agree with and support our preliminary study in which combinations of QUER at different doses reduced the response of another important clinical NSAID, diclofenac (DIC), in rats [16].

In our preliminary study, we hypothesized that the presence of QUER produced a reduction in the antinociceptive effect of DIC through a pharmacokinetic influence since QUER is an inhibitor of cytochrome P450 subtype 2C9, which metabolizes DIC by decreasing the concentration of 4-hydroxy diclofenac, a bioactive metabolite of DIC [38]. It is known that the co-administration of drugs could elicit pharmacokinetic and/or pharmacodynamic interactions. With respect to this, the NSAIDs (IND, KET, and CEL) used in this study do not produce active metabolites [39,40,41] as DIC does. Therefore, a pharmacokinetic process (specifically in the metabolism phase), if any, might not be the main reason for the negative interaction between QUER and the NSAIDs explored in this study. On the other hand, in silico pharmacokinetic predictions revealed that QUER exhibits high gastrointestinal absorption but low blood–brain barrier permeability and presents a high polar surface area (TPSA = 131.36 Å^2^), which may limit tissue penetration. Although not a P-glycoprotein substrate, QUER inhibits multiple cytochrome P450 enzymes (CYP1A2, CYP2D6, and CYP3A4), suggesting the potential for drug–drug interactions [42]. The low LogP and moderate bioavailability score (0.55) are consistent with its known rapid clearance and poor systemic exposure in vivo, suggesting that not only pharmacodynamic but also pharmacokinetic processes might influence the drug interactions of QUER and NAIDs, and this will be interesting to explore in the future. Regarding a possible pharmacokinetic interaction in the absorption process of QUER and NSAIDs, since these drugs are absorbed in the stomach via passive diffusion [39,40,41,42], they were administered via different routes of administration, for example, ip vs. po.

Regarding the pharmacodynamic interaction, it is known that a combination of two or more drugs acting via a similar mechanism of action could promote the antagonistic response of the combined drugs, in part due to saturation of the same pathway [43]. In this sense and according to the molecular docking, all the NSAIDs explored in this study and QUER share similar capability to interact with both COX-1 and COX-2 isoenzymes, suggesting that this can be one of the mechanisms of action involved in their negative pharmacological interaction. The inhibition constants for IND, KET, and CEL based on the COX-2 isoenzyme are 0.15 µM, 0.4 µM, and 5 µM, respectively [44,45], while for QUER, it is 10.13 µM [46]. In addition, it has been demonstrated that QUER inhibits the activation of the phosphoinositide 3-kinase (PI3K) pathway, reducing the expression and production of COX-2, as is performed by NSAIDs [47]. The differential affinity of these drugs for COX-2 might be involved in the differential responses of QUER and the evaluated NSAIDs in this study. However, other mechanisms might be participating; QUER inhibits cytokine production in inflammatory pain [13,14] like several NSAIDs do, and in addition, similar or contrasting interactions can be possible depending on the kind of analgesic drug or if pain is acute or chronic; for example, QUER enhanced the antinociceptive effect of a sigma-1 receptor antagonist (BD-1063) in a neuropathic pain model [48].

Although QUER exhibited a high binding affinity toward COX-2 (−9.2 kcal/mol), comparable to that of the clinically effective inhibitor CEL (−9.5 kcal/mol), its lack of COX-2 inhibition in vivo suggests that high docking affinity does not necessarily translate into functional inhibition. Several mechanistic hypotheses may explain this apparent discrepancy: first, allosteric modulation may be involved. QUER could bind to a peripheral or alternative pocket on COX-2, distinct from the catalytic site, leading to minimal or no interference with arachidonic acid conversion. This non-orthosteric binding might still be scored favorably in docking simulations, particularly when flexible side-chain rearrangements or solvent effects are not considered. Structural studies have shown that COX-2 possesses potential allosteric sites capable of accommodating small ligands, which may modulate enzyme activity without full inhibition [49]. Second, functional selectivity (biased inhibition) may play a role. Polyphenols like QUER are known to exert diverse biological effects, including modulation of the redox status, cytokine production, and transcriptional regulation of inflammatory mediators. These indirect actions may not require the direct enzymatic inhibition of COX-2 and could explain their variable performance in biochemical versus cellular or in vivo assays [50]. Third, it is essential to consider that pharmacokinetic (PK) limitations may prevent QUER from reaching effective concentrations at the target site in vivo. QUER is known to have poor oral bioavailability, extensive first-pass metabolism, and rapid clearance due to glucuronidation and sulfation. These factors severely limit its systemic exposure and target engagement, despite favorable in silico profiles [51]. In contrast, celecoxib exhibits excellent absorption, plasma stability, and tissue penetration, which contribute to its clinical efficacy [40]. This highlights the importance of integrating pharmacokinetic studies (e.g., ADME profiling, metabolite identification, and plasma concentration–time curves) to distinguish between pharmacodynamic failure (e.g., ineffective binding or non-inhibitory binding) and pharmacokinetic constraints. Further studies, including enzyme kinetics, site-directed mutagenesis, and molecular dynamic simulations, are warranted to clarify whether quercetin’s interaction with COX-2 is allosteric, transient, or simply non-productive under physiological conditions.

The results of this study are relevant for pain therapy since QUER is recommended as a nutraceutical product because of its potential antioxidant and anti-inflammatory activities [51], and this kind of product is chronically used as a supplement to the drugs conventionally used in chronic diseases as gouty arthritis, without consideration that their interactions are not always adequate or beneficial. In this study, after the acute or repeated administration of combined QUER and NSAIDs in the PIFIR model of arthritis gout-like pain, the antinociceptive response was reduced. However, the anti-inflammatory effect of these analgesic drugs was not modified, as observed in the carrageenan-induced edema test, suggesting that more than one mechanism of action might be involved in the infra-additive effects observed in this investigation. It is important to mention that in our study, no adverse effects were observed following the daily administration of QUER (100 mg/kg, orally) for 10 days in rats. This information agrees with a previous study analyzing QUER based on behavior and metabolic processes with the sub-chronic administration of 250 mg/kg/day for 14 days, which reported no toxicity or histological changes in mice [52]. The acute toxicity of QUER has only reported after high doses such as 1500 and 2000 mg/kg, which significatively increase biomarkers of hepatotoxicity induced by oxidative stress, in addition to decreasing the expression of tumor suppressor genes, considering that high doses of QUER could be carcinogenic [53]. Likewise, the chronic administration of QUER for 2 years at 1900 mg/kg/day reduced body weight gains in the last year of the study, with the presence of neoplastic lesions in the kidney of rats, including nephropathy, hyperplasia, and neoplasia of the renal tubular epithelium [54].

The strengths of this study are that the analysis of the antinociceptive effect was performed similarly to pain induced after a noxious or pathological stimulus, such as the accumulation of uric acid crystals in gouty arthritis. Most studies of the antinociceptive effects of drugs are carried out with the administration of the treatments before inducing the noxious stimulus. On the other hand, the limitation of this study is that our experimental design did not explore pharmacodynamic or pharmacokinetic mechanisms. It would be interesting to explore them in the future.

## 4. Materials and Methods

### 4.1. In Vivo Study

#### 4.1.1. Animals

The experiments were performed using 384 male Wistar rats (180–200 g) from the bioterium of the Facultad de Medicina at the Universidad Nacional Autónoma de México (UNAM). Animals were divided into groups of 6 individuals and housed in standard clear plastic cages with access to water and food ad libitum, standard conditions of controlled temperature (22 ± 1 °C) and humidity (60–70%), and under 12 h light–dark cycles. Rats were allowed five days to adapt to their environment before initiation of the experiments. Twelve hours before the experiments, food was removed, but free access to water was maintained. Experiments were performed between 7:00 a.m. and 3:00 p.m. All experiments followed the national guidelines for technical specifications for the production, care, and use of laboratory animals (NOM-062-ZOO, 1999) [55] and the ARRIVE 2.0 guidelines [56], as well as ethical guidelines for experimental research involving conscious animals [57]. This protocol was approved by the Institutional Committees of Ethics, Research, and Care and Use of Laboratory Animals of the Facultad de Medicina at UNAM (FM/DI/054/2018). Each animal was used only once and euthanized at the end of the experiments via cervical dislocation from previous anesthesia. All efforts were made to minimize the number of animals used and their suffering.

#### 4.1.2. Compounds

Quercetin (QUER), indomethacin (IND), celecoxib (CEL), uric acid, and λ-carrageenan were purchased from Sigma-Aldrich (St. Louis, MO, USA). Ketorolac (KET) was purchased from Cayman Chemical (Ann Arbor, MI, USA). A suspension of uric acid (30%) was prepared with mineral oil and used to induce gout-like pain in the rats. A solution of λ-carrageenan (1%) was prepared with a 0.9% saline solution (SS) and used to induce inflammation in the rats. QUER was prepared with 0.5% Tween 80 in SS, CEL with 0.5% methylcellulose in SS, IND with 1% sodium carbonate in SS, and KET with SS alone. All drugs were freshly prepared on the day of the experiments and administered systemically at a volume of 10 mL/kg, except uric acid and carrageenan, which were locally administered. Solvents are known to play a key role in drug solubility, which in turn improves bioavailability and the therapeutic response by enhancing absorption. In our study, QUER, as a pure compound, was poorly soluble in water; therefore, a surfactant such as Tween 80 was used as a vehicle in a 0.5% saline solution to improve QUER’s solubility. Additionally, a group administrated with this vehicle was included in the study to verify that it does not produce antinociceptive or anti-inflammatory effects per se.

#### 4.1.3. Nociceptive and Inflammatory Test

##### Pain-Induced Functional Impairment in the Rat (PIFIR) Model

To induce gout-like pain in the animals, the pain-induced functional impairment in the rat (PIFIR) model was employed [24]. An injection of 50 µL of 30% uric acid was applied into the knee joint of the right hind limbs of the rats. After that, one electrode was placed on the plantar surface of each hind limb of the animals to be connected to an interface (OmniAlva OASDA System) to record the contact time of each paw on the cylinder surface. Subsequently, the animals were forced to walk at 4 rpm for 2 min every 30 min to assess the functionality of their limbs. The percentage of functionality index (% FI) was calculated with the contact time of the injured paw (administered with uric acid) divided by the contact time of the uninjured paw (paw without uric acid), multiplied by 100. Approximately 2 h after the administration of uric acid, the animals developed dysfunction in the injured paw, reflected by the functionality of zero or less than 10%. Then, the treatments under evaluation must be administered [24]. The recovery of limb functionality induced by the treatments was interpreted as the antinociceptive effect.

##### Carrageenan-Induced Paw Edema Model in Rats

The anti-inflammatory effect of the drugs was determined by performing the carrageenan-induced hind paw edema test. In this model, 100 µL of 1% λ-carrageenan was subcutaneously injected in the plantar surface of the right hind paws of rats [58]. Carrageenan induced an increase in the paw volume (edema) of each animal over time. The paw volume (mL) was measured using a plethysmometer instrument (Mod. 7150 Ugo Basile). Measurements of the right paw of each rat were made earlier than the induction of edema (before subplantar carrageenan injection, baseline volume) and each hour for 6 h after carrageenan injection. The percentage of the increase in the paw volume was calculated to assess the intensity of edema. The baseline paw size of each animal was considered 0%. To determine the anti-inflammatory effect, treatments were administered 30 min before carrageenan-induced paw edema and were compared to the VEH group.

#### 4.1.4. Experimental Design

##### Antinociceptive Effect of QUER, IND, KET, and CEL Alone and in Combination Evaluated Using the PIFIR Model

In the first set of experiments, the individual antinociceptive effect of IND (1–10 mg/kg), KET (0.18–10 mg/kg), or CEL (1–31.6 mg/kg), administered via an oral route (po), and QUER (31.6–316.2 mg/kg), administered intraperitoneally (ip), was determined in independent groups of rats. The doses of the drugs were selected considering previous studies [24,59,60] and to build the dose–response curves with a minimum number of doses, with the advantage of a logarithmic increase (quarter or half) between doses to cover the range of a window of biological activity from the threshold to the maximum effect of each drug.

In a second set of experiments, IND (1–10 mg/kg), KET (0.31–10 mg/kg), or CEL (1–31.6 mg/kg) was administered in combination with QUER (31.6 or 100 mg/kg) resulting in ten combinations of IND + QUER, eleven combinations of KET + QUER, and eight combinations of CEL + QUER to determine the SSI [17]. Each dose of the drugs, administered individually and/or in combination, was given to 6 animals approximately 2 h after the uric acid administration. The antinociceptive effect of each treatment was determined every 15 min for 1 h and every 30 min for the next 3 h. Animals that did not develop dysfunction of less than 10% after the administration of uric acid were excluded. Subsequently, through an SSI analysis, the type of antinociceptive interaction produced by each combination was determined, and the combination of each NSAID with QUER that induced the maximum antinociceptive interaction (MAI) was selected for the following experiments. Forty-eight groups of animals were used to determine the antinociceptive effect of different treatments alone or in combination, including one group with the vehicle (negative control). In this part of the study, 288 animals were used.

To determine if the subchronic administration of QUER continued to reduce the antinociceptive effect of NSAIDs, as the acute administration explored, six groups received different pretreatments 10 days before the PIFIR model. Three groups of them received the daily administration of VEH (0.5% Tween 80 in SS, po) or QUER (100 mg/kg, po). On the 11th day, rats in the six groups were intraarticularly administered 30% uric acid to induce gout-like pain. Two hours after nociception induction, rats in one group of each pretreatment (VEH or QUER) were administered a single dose of IND (10 mg/kg), KET (10 mg/kg), or CEL (10 mg/kg), po. Then, the functionality index (FI) was determined every 30 min for 4 h. A group treated with QUER (100 mg/kg/day for 10 days) or QUER (100 mg/kg, po) based on acute administration was used as the controls. In this part of the study, 48 animals were used.

##### Anti-Inflammatory Effect of IND, KET, or CEL Combined with QUER and Evaluated Based on Carrageenan-Induced Edema in Rats

To corroborate the anti-inflammatory effect, each combination inducing the maximal antinociceptive interaction (MAI) and the corresponding doses in the individual administration were assessed using the carrageenan-induced edema test using independent groups. In this part of the study, 48 animals were used, divided into eight groups of 6 rats each that were administered treatments 30 min before the subcutaneous injection of carrageenan (1%, 100 µL) in the plantar surfaces of the right hind paws of rats [58]. The percentage of edema in the paw of each animal was calculated with the following formulae:

$$\%Paw\;edema=\frac{\left(Vt-Vo\right)}{Vo}\times 100$$

where Vt is the rat paw volume at time “t” and Vo is the initial rat paw volume (before carrageenan injection).

A diminution of the carrageenan-edema induced by the treatments in comparison to the VEH group was considered an anti-inflammatory effect.

#### 4.1.5. Statistical Analysis

The sample size for each group was estimated considering the difference between a value that could represent a minimal effect with clinical relevance of 20% (µ_2_) of an antinociceptive or anti-inflammatory effect minus a value that represents the lack of relief of 5% (µ_1_), the representation of the variability that we expected to find between subjects with the same treatment (SD = 9), a significance level of 95% (Z_α/2_ = 1.96), and a statistical power of 80% (Zβ = 0.84). With the following formula, an “n” of 5.6 was calculated; therefore, six animals were used per experimental group [61].

$$n=\frac{2\cdot {SD}^{2}\cdot {\left(Z_{\alpha /2}+Z\beta \right)}^{2}}{{\left({µ}_{2}-{µ}_{1}\right)}^{2}}$$

The antinociceptive and anti-inflammatory effects of treatments in individual or combined administration were reported as the mean ± standard error of the mean (SEM) of 6 animals per treatment. The AUC for each treatment was calculated from the respective temporal courses using the trapezoidal method [62] to build the corresponding dose–response curves (DRC).

The antinociceptive interaction between QUER and IND, KET, or CEL was analyzed using the surface of synergistic interaction (SSI) method [53]. This method consists of building three-dimensional graphs for each combination. In the first set of graphs, the DRCs of the respective NSAIDs (IND, KET, or CEL) are represented on the “X” axis; the antinociceptive effect is shown on the “Y” axis and expressed as the AUC, while the DRC of QUER is presented on the “Z” axis. Each intermediate point represents the antinociceptive effect obtained for each combination. In the second set of graphs, the “Y” axis shows the “difference” of the experimentally obtained effect with each combination minus the sum of the individual effect of each drug of the respective combination. Finally, in the third set of graphs of each combination, the SSI obtained by joining the points graphed in the second set of graphs is shown. The type of interaction resulting from the combination of several doses of two drugs, as well as the prediction of the expected effect with the combination of two drugs that were not tested experimentally, is detected with this method. In this regard, if the points of SSI are above the zero value on the horizontal axis, the interaction is considered supra-additive; if the points of SSI are in the zero value, the interaction is additive; while if the points are below the zero plain, the interaction is considered infra-additive. With this analysis, it is possible to determine the combination that induces the MAI.

Statistical differences in treatments were obtained through a one-way or two-way analysis of variance (ANOVA) for the independent samples and multiple comparisons, followed by Tukey’s post hoc tests according to the comparisons. A Student’s *t*-test was also used when two groups were compared. In all the statistical analyses, a *p* value < 0.05 was considered significant. All comparisons were carried out using GraphPad Prism 8.0 software.

### 4.2. Molecular Docking (In Silico) Study

#### 4.2.1. Ligand and Receptor Preparation

The three-dimensional structures of cyclooxygenase-1 (COX-1) and cyclooxygenase-2 (COX-2) were retrieved from the Protein Data Bank (PDB) under accession codes 6Y3C and 8ET0, respectively. The chemical structures of the ligands QUER, CEL, IND, and KET were downloaded from the PubChem database in SDF format. Ligand structures were subjected to geometric optimization and energy minimization using Avogadro software, applying the Merck Molecular Force Field 94 (MMFF94). The resulting minimized energies (expressed in kilojoules per mole, kJ/mol) were as follows: QUER, 217.297 kJ/mol; CEL, 251.44 kJ/mol; IND, 543.782 kJ/mol; KET, 4.55 kJ/mol. These values reflect the relative conformational stability of each ligand before docking simulations.

All ligand files were converted to PDBQT format using AutoDock Tools, while receptor structures were prepared using the DockPrep module in UCSF Chimera, including the remotion of water molecules, addition of polar hydrogens, and assignment of Gasteiger charges.

#### 4.2.2. Molecular Docking Simulations

Molecular docking simulations were conducted using AutoDock Vina to evaluate the binding affinities and interaction profiles of ligands with COX-1 and COX-2. Grid boxes were defined to encompass the catalytic domains of each receptor, ensuring thorough coverage of the active site. The exhaustiveness parameter was set to 8, and five binding poses were generated for each ligand–target complex. The pose with the lowest binding energy (kcal/mol) was selected for the interaction analysis.

#### 4.2.3. Interaction Analysis

The ligand–receptor complexes were analyzed using BIOVIA Discovery Studio Visualizer. Three-dimensional (3D) and two-dimensional (2D) interaction maps were generated to identify the nature of key non-covalent interactions, including conventional hydrogen bonds, van der Waals contacts, π-π stacking, and electrostatic interactions. Residues participating in each interaction were recorded and classified accordingly.

## 5. Conclusions

In conclusion, although QUER did not modify the anti-inflammatory effect of the assessed NSAIDs, the infra-additive response caused by the antinociceptive effects of IND, KET, and CEL in gouty arthritic-like pain in the presence of this flavonoid suggest that their combination is not convenient as an alternative in the treatment of this kind of pain. Thus, patients who use some NSAIDs for the relief of inflammatory pain should be cautious in the use of dietary supplements containing QUER. More studies, not only about pharmacodynamics but also pharmacokinetics, are required to clarify the possible mechanisms involved in these interactions, but the preclinical evidence of this study does not recommend their simultaneous use.

## Figures and Tables

**Table 1:** Antinociceptive effect of NSAIDs in rats with gout-like pain combined with repeated administration (RA) of QUER (100 mg/kg/day for 10 days).

Group	Antinociceptive Effect (%)							
	0.5 h	1 h	1.5 h	2 h	2.5 h	3 h	3.5 h	4.0 h
SS + QUER-RA	36.6 ± 14.4	39.5 ± 19.1	37.6 ± 19.3	39.1 ± 18.5	35.3 ± 13.8	36.0 ± 16.0	37.1 ± 11.1	35.0 ± 13.1
SS + VEH-RA	1.9 ± 1.6 *	2.7 ± 2.7 *	3.6 ± 2.6 *	1.3 ± 0.9 *	4.7 ± 2.7 *	4.1 ± 2.7 *	3.7 ± 1.8 *	2.4 ± 1.8 *
IND (10 mg/kg) + VEH-RA	42.3 ± 13.5	45.3 ± 14.1	60.7 ± 15.5	56.6 ± 11.9	58.5 ± 12.5	81.9 ± 7.7	66.6 ± 17.2	71.6 ± 10.2
IND + QUER-RA	53.2 ± 14.8	41.2 ± 9.5	40.7 ± 6.6	37.3 ± 5.5	43.2 ± 13.7	68.4 ± 21.7	64.5 ± 11.4	55.7 ± 6.1
KET (5.6 mg/kg) + VEH-RA	38.2 ± 14.3	63.8 ± 15.5	55.9 ± 8.5	59.8 ± 18.7	51.2 ± 16.7	56.8 ± 14.5	52.9 ± 15.4	67.0 ± 23.3
KET + QUER-RA	26.3 ± 10.5	31.1 ± 9.8	39.7 ± 13.1	38.0 ± 11.7	26.4 ± 11.4	32.1 ± 10.6	45.0 ± 16.4	38.0 ± 16.6
CEL (10 mg/kg) + VEH-RA	25.7 ± 6.9	34.6 ± 8.4	45.3 ± 17.4	49.1 ± 13.5	39.4 ± 9.6	45.3 ± 7.9	49.7 ± 10.7	40.7 ± 13.0
CEL + QUER-RA	39.8 ± 14.2	39.2 ± 11.5	58.6 ± 12.8	51.6 ± 10.7	52.5 ± 12.1	59.6 ± 13.3	62.8 ± 10.6	72.1 ± 13.0

Mean ± SEM (n = 6). * *p* < 0.05 vs. SS + QUER (RA), two-way ANOVA followed by Tukey’s test. Saline solution (SS), vehicle (VEH), celecoxib (CEL), indomethacin (IND), ketorolac (KET), quercetin (QUER), repeated administration (RA), non-steroidal anti-inflammatory drugs (NSAIDs).

**Table 2:** Anti-inflammatory effect of NSAIDs in the absence or the presence of QUER in the carrageenan-induced edema test in rats.

Group	Dose (mg/kg)	Paw Edema (%)					
		1 h	2 h	3 h	4 h	5 h	6 h
VEH		25.3 ± 4.4	48.8 ± 3.8	58.6 ± 6.3	61.8 ± 6.2	65.3 ± 6.1	65.3 ± 7.1
QUER	100	31.3 ± 3.2	40.9 ± 5.2	52.4 ± 6.0	58.2 ± 4.0	58.3 ± 2.6	60.5 ± 2.9
IND	10	17.8 ± 2.8	18.8 ± 4.3 *	24.7 ± 3.3 *	28.6 ± 4.1 *	30.1 ± 5.3 *	33.5 ± 7.4 *
IND + QUER		16.1 ± 3.5	18.6 ± 4.9 *	24.3 ± 3.8 *	33.0 ± 5.2 *	38.3 ± 7.1 *	41.8 ± 6.8 *
KET	5.6	25.2 ± 1.7	28.3 ± 2.9 *	29.8 ± 3.3 *	34.3 ± 4.0 *	40.9 ± 5.2 *	45.8 ± 6.0 *
KET + QUER		18.7 ± 2.9	22.4 ± 2.7 *	23.9 ± 4.1 *	29.5 ± 3.7 *	37.3 ± 4.4 *	38.6 ± 4.6 *
CEL	10	21.5 ± 2.5	31.1 ± 3.9 *	38.5 ± 5.5 *	51.5 ± 8.9	55.3 ± 6.6	56.6 ± 7.9
CEL + QUER		24.9 ± 3.8	28.9 ± 4.9 *	34.2 ± 4.3 *	41.5 ± 5.1 *	43.3 ± 5.6 *	44.8 ± 5.6 *

* *p* < 0.05 vs. VEH, two-way ANOVA followed by Tukey’s test.

## Data Availability

The datasets generated and/or analyzed during the current study are available from the corresponding author upon reasonable request.

## Supplementary Materials

The following supporting information can be downloaded at: https://www.mdpi.com/article/10.3390/molecules30153196/s1, Figure S1: Effect of QUER (100 mg/kg) in acute or sub-chronic administration (for 10 days) after 2.5 h of the intra-articular administration of uric acid (30%) on the functionality index (%) of rats.

## Abbreviations

The following abbreviations are used in this manuscript:

ANOVA	Analysis of Variance
AUC	Area Under the Curve
CEL	Celecoxib
DRC	Dose–Response Curve
IND	Indomethacin
KET	Ketorolac
NSAIDs	Non-Steroidal Anti-Inflammatory Drugs
PIFIR	Pain Induced Functional Impairment Model in Rats
QUER	Quercetin
SEM	Standard Error of Mean
SS	Saline Solution
SSI	Surface of Synergistic Interaction
VEH	Vehicle
