# Supplementary material for: Quercetin Reduces Antinociceptive but Not the Anti-Inflammatory Effects of Indomethacin, Ketorolac, and Celecoxib in Rats with Gout-like Pain
Source: Molecules. 2025 Jul 30;30(15):3196. doi: 10.3390/molecules30153196 (PMC12348991; doi:10.3390/molecules30153196)
Supplement: Supplementary file 1 [file molecules-30-03196-s001.zip › molecules-3678016-supplementary.pdf]

Quercetin reduces antinociceptive but not the anti-inflammatory effects of indomethacin, ketorolac, and celecoxib in rats with gout-like pain

José Aviles-Herrera[1], Guadalupe Esther Ángeles-López[1*], Myrna Déciga-Campos[2], María Eva González Trujano[3], Gabriel Fernando Moreno-Pérez[3], Ricardo Reyes-Chilpa[4], Irma Romero[5], Amalia Alejo-Martínez[6], Rosa Ventura-Martínez[1*]

[1]Departamento de Farmacología, Facultad de Medicina, Universidad Nacional Autónoma de México (UNAM), Ciudad de México, México.
[2]Sección de Estudios de Posgrado e Investigación, Escuela Superior de Medicina, Instituto Politécnico Nacional (IPN), Ciudad de México, México.
[3]Laboratorio de Neurofarmacología de Productos Naturales, Dirección de Investigaciones en Neurociencias, Instituto Nacional de Psiquiatría "Ramón de la Fuente Muñiz", Ciudad de México, México.
[4]Instituto de Química, Universidad Nacional Autónoma de México (UNAM), Ciudad de México, México.
[5]Departamento de Bioquímica, Facultad de Medicina, Universidad Nacional Autónoma de México (UNAM), Ciudad de México, México.
[6]Laboratorio 7 de Dolor y analgesia. Departamento de Farmacobiología. Centro de Investigación y de Estudios Avanzados (CINVESTAV), Sede sur, Ciudad de México, México.

*Corresponence: rventuram@comunidad.unam.mx; rventuram7@hotmail.com (RVM); guadalupe@facmed.unam.mx (GEAL).

Supplementary material

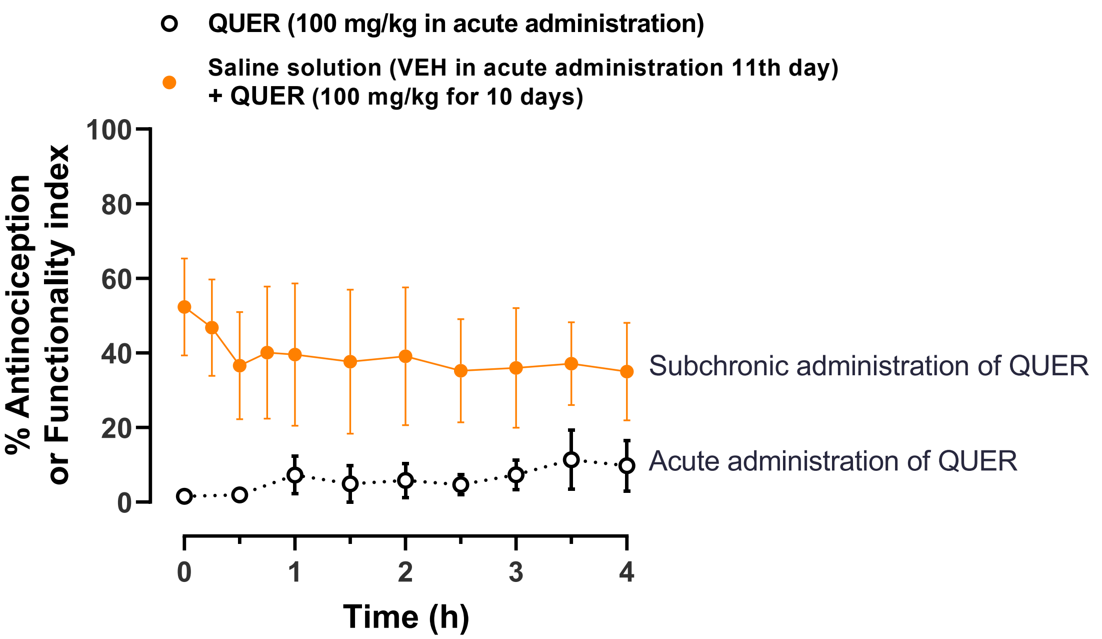

Figure S1. Effect of QUER (100 mg/kg) in acute or sub-chronic administration (for 10 days) after 2.5 hours of the intra-articular administration of uric acid (30%) on the functionality index (%) of rats.
